# The WAVE complex in developmental and adulthood brain disorders

**DOI:** 10.1038/s12276-024-01386-w

**Published:** 2025-01-07

**Authors:** Hyung-Goo Kim, Clara Berdasco, Angus C. Nairn, Yong Kim

**Affiliations:** 1 Department of Neurosurgery, Robert Wood Johnson Medical School, Rutgers University, Piscataway, NJ 08854 USA; 2 Department of Psychiatry, Yale School of Medicine, Connecticut Mental Health Center, New Haven, CT USA; 3 Brain Health Institute, Rutgers University, Piscataway, NJ 08854 USA

**Keywords:** Autism spectrum disorders, Actin

## Abstract

Actin polymerization and depolymerization are fundamental cellular processes required not only for the embryonic and postnatal development of the brain but also for the maintenance of neuronal plasticity and survival in the adult and aging brain. The orchestrated organization of actin filaments is controlled by various actin regulatory proteins. Wiskott‒Aldrich syndrome protein-family verprolin-homologous protein (WAVE) members are key activators of ARP2/3 complex-mediated actin polymerization. WAVE proteins exist as heteropentameric complexes together with regulatory proteins, including CYFIP, NCKAP, ABI and BRK1. The activity of the WAVE complex is tightly regulated by extracellular cues and intracellular signaling to execute its roles in specific intracellular events in brain cells. Notably, dysregulation of the WAVE complex and WAVE complex-mediated cellular processes confers vulnerability to a variety of brain disorders. De novo mutations in WAVE genes and other components of the WAVE complex have been identified in patients with developmental disorders such as intellectual disability, epileptic seizures, schizophrenia, and/or autism spectrum disorder. In addition, alterations in the WAVE complex are implicated in the pathophysiology of Alzheimer’s disease and Parkinson’s disease, as well as in behavioral adaptations to psychostimulants or maladaptive feeding.

## WAVE proteins link actin dynamics to brain disorders

The WAVE complex is a group of proteins that work together to regulate actin, an essential component of the cell cytoskeleton. This article reviews how changes in the WAVE complex can affect brain function and contribute to various brain disorders. It summarizes how different parts of the WAVE complex interact with other proteins to regulate actin polymerization. This regulation is crucial for programming brain cell growth, migration, and synaptic formation and function. This article also explores recent studies on genetic changes in the WAVE complex and their links to developmental and adult brain disorders. Key findings suggest disruptions in the WAVE complex can lead to conditions like intellectual disabilities, autism, and neurodegenerative diseases such as Alzheimer’s.

This summary was initially drafted using artificial intelligence, then revised and fact-checked by the author.

## Introduction

Actin polymerization is an ATP-dependent spontaneous process that can be initiated by the activation of the actin-related protein (ARP) 2/3 complex^1,2^. The rate-limiting step in initiating actin polymerization is the formation of dimers or trimers of actin. The ARP2/3 complex is composed of seven subunits, and the activated ARP2 and 3 subunits mimic the dimer of actin, thereby initiating de novo actin polymerization. Because the ARP2/3 complex can bind to preexisting filamentous actin (F-actin), it can initiate the formation of a branched form of F-actin^2,3^. In the brain, branched F-actin plays a critical role in neurons, including anchoring synaptic vesicles at presynaptic terminals, inducing dynamic morphological changes in dendritic spines, and scaffolding postsynaptic proteins. Notably, process extension in oligodendrocytes requires ARP2/3-dependent actin polymerization, but actin disassembly is a prerequisite for axon myelination^4^. Dynamic morphological changes in astrocytes and microglia are mediated by ARP2/3-mediated actin polymerization^5,6^.

**Fig. 1:**
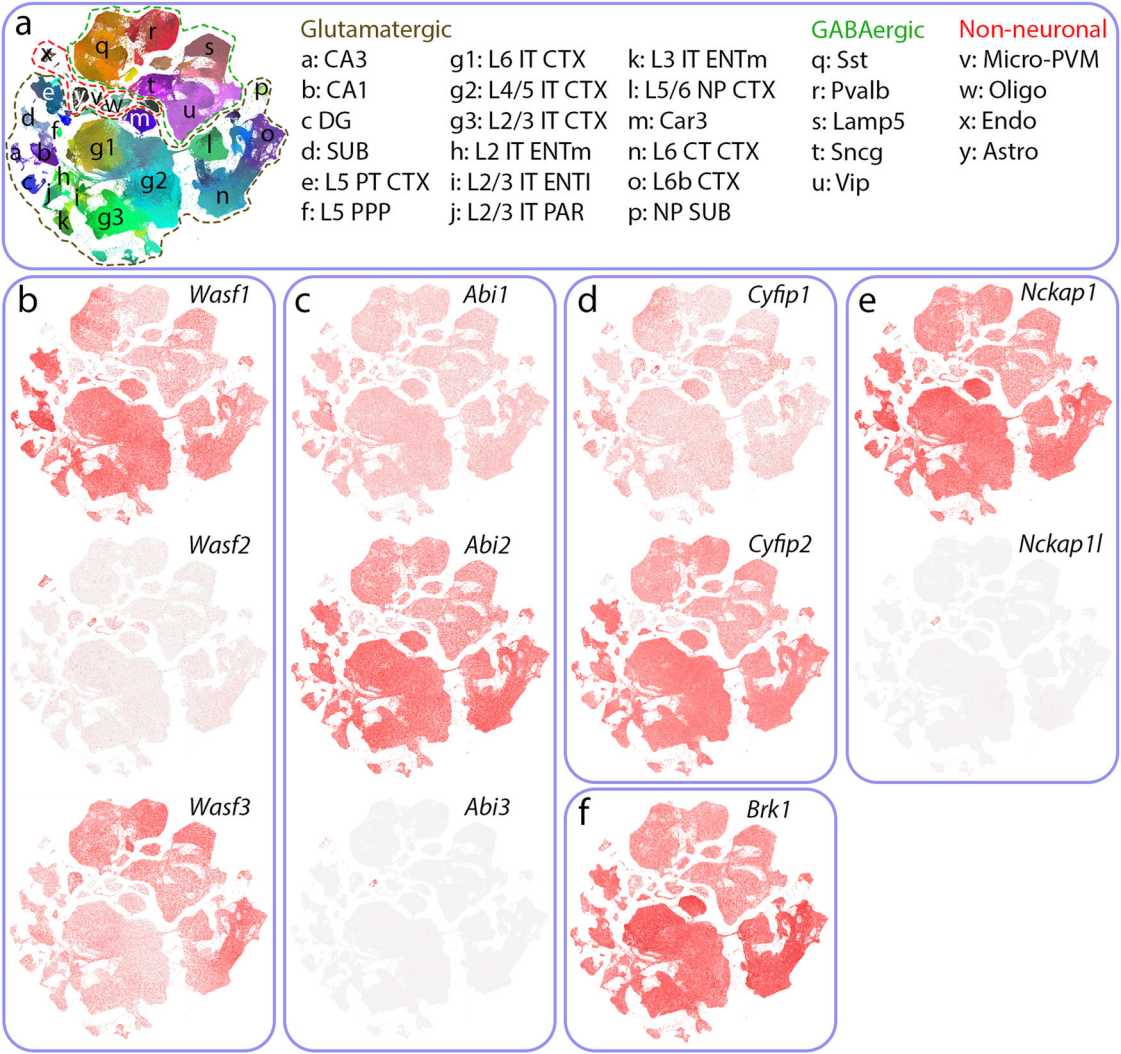
Expression pattern of subunit paralogs of the WAVE complex. **a** Scatter plot of single-cell RNA-seq data for single cells isolated from the whole hippocampus and cortex (left panel). The glutamatergic, GABAergic, and non-neuronal cell types corresponding to the segregated cells are indicated (right panel). Publicly available single-cell RNA-seq data for WAVE genes: *Wasf1*, *2* and *3* (**b**), *Abi1*, *2* and *3* (**c**), *Cyfip1* and *2* (**d**), *Nckap1* and *1l* (**e**) and Brk1 (**f**). Allen Institute for Brain Science (https://portal.brain-map.org/atlases-and-data/rnaseq).

The ARP2/3 complex is activated by nucleation-promoting factors^2^. Wiskott‒Aldrich syndrome protein (WASP) and WASP-family verprolin-homologous protein (WAVE) belong to the WASP family, which are the most well understood nucleation-promoting factors and consist of WASP/N-WASP, Scar/WAVE1-3, WASH, JMY, WHAMM and WHIMP proteins^7,8^. Three paralogs of WAVE (WAVE1-3; gene names: *Wasf1*, *Wasf2* and *Wasf3*, respectively) are preferentially but not exclusively expressed in distinct brain cell types (Fig. 1 and Table 1). WAVE1 is a brain-enriched regulator that is especially abundant in neurons^9–12^, oligodendrocyte precursor cells and mature oligodendrocytes^13^. WAVE2 is expressed at high levels in microglia and vascular endothelial cells^14,15^. WAVE3 is also expressed mainly in the brain^16^, where its levels in interneurons and astrocytes are relatively high^17,18^. WAVE proteins exist as heteropentameric protein complexes, termed the WAVE complex^19,20^ or WAVE regulatory complex^21^, together with NCK-associated protein 1 (NCKAP1) or NCK-associated protein 1-like (NCKAP1L), cytoplasmic FMR1 interacting protein (CYFIP) 1 or 2, ABL-interactor (ABI) 1, 2 or 3, and BRK1 (also called HSPC300)^11,19,22,23^. Compared with their paralogs, WAVE1, CYFIP2, NCKAP1 and ABI2 are relatively abundant in the brain^19,20,22^ (Fig. 1). The C-terminal WASP homology 2 (WH2)-central-acidic (WCA) domain of WAVE binds to and activates the ARP2/3 complex^24,25^, whereas CYFIP, NCKAP, ABI and BRK1 are involved in the regulation of WAVE complex formation, stability, subcellular localization or binding to upstream ligands or activators^21,26–28^. Like WAVE proteins, paralogous members of each subunit are preferentially but not exclusively expressed in specific cell types in the brain (Fig. 1 and Table 1). Promiscuous interactions among different paralogous subunits of the pentameric complex likely exist, increasing regulatory and functional diversity, although no specific rule for the interaction between paralogs of subunits has been established.

**Table 1:** Relative expression of the subunit paralogs of the WAVE complex in brain cell types.

The WAVE complex	Glutamatergic neurons	GABAergic neurons	Microglia/PVM	Oligo-dendrocytes	Astrocytes	Endothelial cells
*Wasf1*	++++	+++	+	++	++	+
*Wasf2*	++	++	++++	+++	+++	++++
*Wasf3*	+++	++++	+	++	++++	+
*Abi1*	+++	+++	++	++	+++	++
*Abi2*	++++	+++	+	++	++	+
*Abi3*	−	−	++++	−	−	+
*Cyfip1*	+++	+++	++++	++	+++	++
*Cyfip2*	++++	+++	+	+++	++	+
*Nckap1*	++++	+++	+	+++	+++	+++
*Nckap1l*	−	−	++++	−	−	−
*Brk1*	++++	+++	+++	+++	+++	+++

Very high expression (++++), high expression (+++), medium expression (++), low expression (+), and negligible expression (−).

*PVM* perivascular macrophages.

In this review, we highlight the roles of the WAVE complex as a cellular activity-dependent actin regulator in the brain. We also review recent human genetic studies and studies using rodents and other model organisms that describe the roles of WAVE complex alterations in the pathophysiology of developmental and adulthood brain disorders.

## The WAVE complex is a cellular activity-dependent actin regulator

**Fig. 2:**
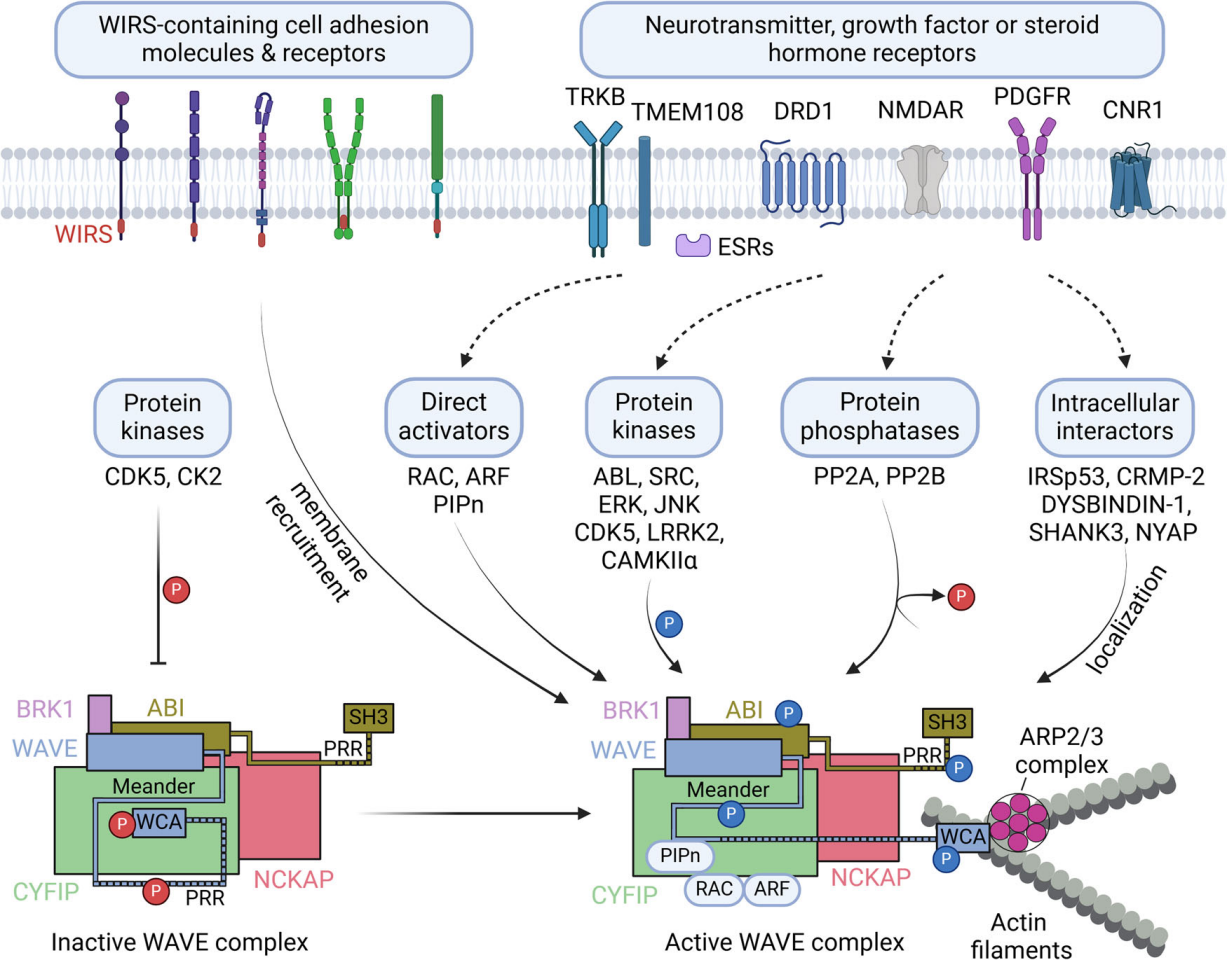
A proposed model for various input signals and regulators converging on the WAVE complex, leading to ARP2/3 complex-mediated actin polymerization. Constitutively active kinases (CDK5 and CK2) basally phosphorylate WAVE, and the intramolecular interaction of the WCA domain with the meander region in WAVE inhibits actin polymerization. In contrast, activated neurotransmitter, growth factor or steroid hormone receptors and their signaling pathways may activate various regulators of the WAVE complex, including direct activators (such as RAC, ARF and PIPn), protein kinases or phosphatases that modulate phosphorylation levels in the WAVE complex, and protein interactors that localize the WAVE complex at locally active sites. WIRS-containing cell adhesion molecules or receptors recruit the WAVE complex to cell membranes. The integration of such input signals results in spatially and temporally orchestrated ARP2/3-mediated actin polymerization in a variety of cellular events. This figure was created with BioRender.com.

Multiple signaling pathways converge on the WAVE complex, resulting in the dynamic regulation of actin polymerization (Fig. 2). Initially, WAVE was identified as a downstream target of Rac, a Rho-family GTPase, and as a mediator of Rac-induced reorganization of the actin cytoskeleton^25,29^. Rac1 binds to two sites on CYFIP in the WAVE complex, leading to WAVE activation^28,30–32^. A proteomic analysis revealed that WRP, a Rac-selective GTPase-activating protein, binds to WAVE1 via its SH3 domain (Src homology domain 3) and inhibits Rac’s ability to regulate WAVE1-mediated actin polymerization^33^. In addition, ARF1 directly binds to a distinct site on CYFIP1, which is greatly enhanced by RAC1 binding, and thereby activates the WAVE complex^34,35^. Acidic phospholipids such as phosphatidylinositol (3,4,5)-triphosphate (PIP_3_) or PI(4,5)P_2_ bind to the basic surface of WAVE2 complexes, enhancing WAVE2 complex activation via prenylated Rac1^20^ (Fig. 2). However, the activation of the native WAVE1 complex isolated from the bovine brain was not affected by prenylated Rac1 or PIP_2_, indicating potential differences in the regulation of the WAVE1 and WAVE2 complexes^20^.

**Fig. 3:**
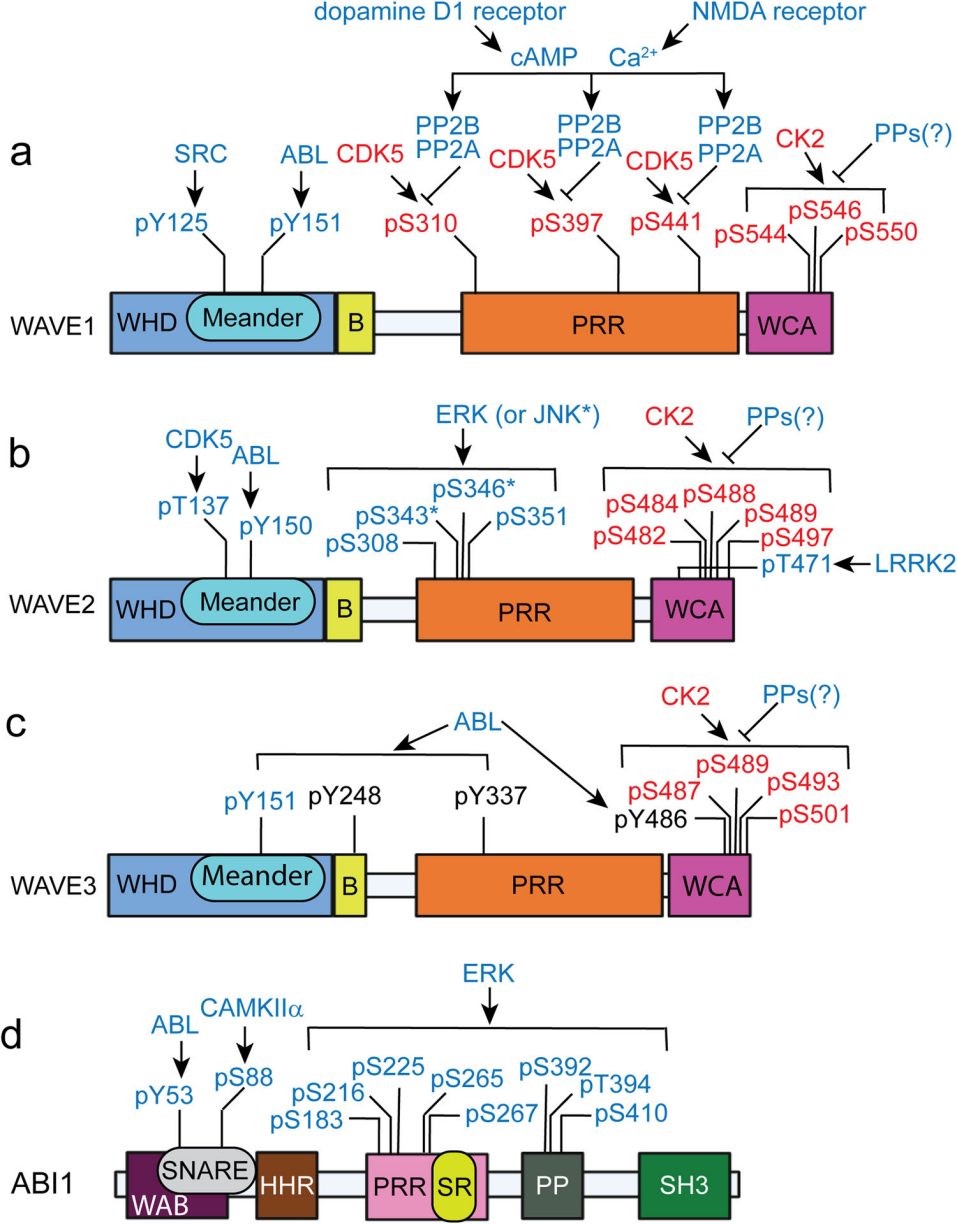
Regulation of the WAVE complex by phosphorylation. Phosphorylation at multiple sites on WAVE (**a**–**c**) and ABI1 (**d**) is regulated by protein kinases or protein phosphatases. The proposed roles of phosphorylation (stimulatory as blue, inhibitory as red, and uncharacterized as black) are indicated. The phosphorylation sites are illustrated for human sequences. WHD WAVE homology domain, B basic domain, PRR proline-rich region, WCA WASP homology 2 (WH2), central, acidic domain, WAB WAVE-binding domain, SNARE soluble N-ethylmaleimide-sensitive factor-activating protein receptor domain, HHR Hox homology region, SR serine/threonine-rich region, PP polyproline region, SH3 Src homology 3 domain.

Phosphorylation of the WAVE complex is another important regulatory mechanism. The phosphorylation of WAVE or ABI mediates the activation or inhibition of the WAVE complex, depending on specific phosphorylation sites^22,36^ (Figs. 2 and 3). The availability of the WCA region of WAVE for ARP2/3 activation is hindered by multiple interactions with both CYFIP and the “meander” region of WAVE^21^ (Fig. 2). The ABL tyrosine kinase phosphorylates Tyr151 of WAVE1, Tyr150 of WAVE2 or Tyr151 of WAVE3^37,38^, and this phosphorylation is suggested to interfere with the interaction between the WAVE meander region and CYFIP, releasing the WCA region for ARP2/3 activation^21^ (Figs. 2 and 3a–c). The SRC nonreceptor tyrosine kinase also phosphorylates WAVE1 at Tyr125, which is anticipated to destabilize the meander region of WAVE and release the WCA region^21,39^. Phosphorylation of WAVE2 at Ser137 by cyclin-dependent kinase 5 (CDK5) in the meander region is required for PDGF-induced oligodendrocyte precursor cell migration in vitro^40^. ABL also phosphorylates additional Tyr sites, including residues 248, 337 and 486 of WAVE3^37,38^. WAVE2 is phosphorylated by extracellular signal-regulated kinase (ERK) or Jun N-terminal kinase (JNK) at Ser308, Ser343, Thr346 and Ser351, and this phosphorylation is necessary for actin polymerization and the migration of cultured cell lines^41–43^. However, these ERK or JNK phosphorylation sites are not present in WAVE1 or WAVE3^36^. Phosphorylation by ABL, SRC and CDK5 in the meander region and ERK phosphorylation in the proline-rich region mediate the WAVE activation required for lamellipodia formation and cell migration^36^, but the roles of phosphorylation at these sites in the function of the WAVE complex in the brain remain to be further established.

The WAVE1 complex was identified from brain lysates as an interactor of CDK5 complexed with p35, an activating subunit of CDK5^22^. CDK5 phosphorylates Ser310, Ser397 and Ser441 within the proline-rich region of WAVE1, but these three sites are not phosphorylated by ERK. CDK5 phosphorylation of the native WAVE1 complex purified from the rat brain or recombinant WAVE1 inhibits ARP2/3-mediated actin polymerization in vitro^22^. Ser310 is the major phosphorylation site, and a Ser310Ala mutation blocks the inhibitory effect of CDK5 phosphorylation on WAVE1-mediated actin polymerization in vitro^22^. Compared with the WT protein, the expression of a Ser310Ala mutant in WAVE1-knockdown neurons had a greater effect on the recovery of dendritic spine morphology, whereas the expression of a phosphomimetic Ser310Asp mutant had little or no effect on the WAVE1 knockdown-induced phenotype^22^. Notably, CDK5 phosphorylates WAVE1 at Ser310, Ser397 and Ser441 with high basal stoichiometry in the intact brain, brain slices or cultured neurons^22^. Upon stimulation of dopamine D1 receptors (DRD1) or NMDA glutamate receptors in brain slices, cAMP and calcium signaling and downstream protein phosphatase-2A (PP2A) and PP2B dephosphorylate WAVE1, indicating the critical involvement of protein phosphatases in neuronal activity-induced WAVE1 activation^44^ (Figs. 2 and 3a). In primary cultured cortical neurons, repetitive depolarization by KCl induces WAVE1 dephosphorylation in an NMDA receptor-dependent manner^45^. In cultured rat cortical neurons, the phosphorylation of WAVE1 at Ser310, Ser397 and Ser441 was acutely increased by estrogen treatment^46^. The increased phosphorylation and recruitment of CDK5 and WAVE1 to the cell membrane were associated with increased actin thickness and membrane protrusions. Interestingly, changes in CDK5-mediated WAVE1 phosphorylation have been proposed as a downstream event of RAC and G protein signaling^46^. Furthermore, alterations in CDK5 phosphorylation of WAVE1 were observed in the brains of a mouse model of Alzheimer’s disease (AD)^47^, as well as in those of cocaine-administered mice^11^.

Casein kinase 2 (CK2) phosphorylates Ser482, 484, 488, 489 and 497 within the acidic region of the WCA domain of WAVE2^48^. The first three serine sites are conserved in all WAVEs: Ser489 is specific to WAVE2, and Ser497 in WAVE2 is conserved in WAVE3 (Ser501)^36^ (Fig. 3a–c). CK2 is a constitutively active kinase that basally phosphorylates sites within acidic regions. Based on a study in Dictyostelium, phosphorylation of the first two CK2 sites (WAVE1-Ser544 and 546, WAVE2-Ser482 and 484, and WAVE3-Ser487 and 489) is proposed to inhibit WCA function via the facilitation of intramolecular interactions with the basic region in WAVE^49^. Thus, CK2 phosphorylation of the WCA domain of WAVEs and/or CDK5 phosphorylation of the proline-rich region of WAVE1 may confer an inhibitory state, whereas protein phosphatase-mediated dephosphorylation of these sites may activate the WAVE complex^22,43,44,48,49^.

ABI is enriched in dendritic spines and localizes to the postsynaptic density (PSD)^50^. Ca^2+^/calmodulin-dependent kinase IIα (CAMKIIα) interacts with ABI1 and phosphorylates Ser88, which may be required for ABI-mediated dendritic spine maturation^51^ (Fig. 3d). ERK also phosphorylates multiple sites in ABI1, including Ser183, Ser216, Ser225, Thr265, Ser267, Ser392, Thr394 and Ser410^41^ (Fig. 3d). Experiments with proteins containing mutations of these sites implicate a role of ABI1 phosphorylation in the release of the WCA domain and subsequent WAVE complex activation^36,41^. Notably, phosphorylation sites, kinases and functional consequences may vary depending on the cell type and cellular conditions^20,41^. Furthermore, the relevance of CK2- or ERK-mediated phosphorylation mechanisms in the brain or brain cells remains to be further established. Other subunit paralogs, including ABI2^20^, ABI3^52,53^ and CYFIP1/2^54^, also appear to be phosphoproteins, but kinases for specific phosphorylation sites and the roles of their phosphorylation in WAVE complex activity or actin polymerization remain to be fully established.

The translocation and membrane recruitment of the WAVE complex are critical for the engagement of WAVE-mediated actin polymerization at specific functional loci. In addition to RAC (or ARF) and acidic phospholipids (PIP_2_ or PIP_3_), many transmembrane cell adhesion molecules and receptors, including protocadherins, ROBOs, netrin receptors, neuroligins, GPCRs and ion channels, may recruit the WAVE complex to membranes via the interaction of CYFIP and ABI with a conserved motif called WIRS (WAVE complex interacting receptor sequence)^23,28^ (Fig. 2). For example, in *C. elegans*, SYG-1, an adhesion molecule, interacts with the WAVE complex through the WIRS motif, and this interaction is required for synaptic F-actin dynamics, synapse formation and axon branching^55^. Several other binding partners of WAVE1, such as collapsin response mediator protein 2 (CRMP-2 or DPYSL2)^56^ or cannabinoid CB1 receptor (CNR1)^57^, WAVE2 interactions with insulin receptor substrate 53, IRSp53 (BAIAP2) or dystrobrevin-binding protein-1 (DYSBINDIN-1 or DTNBP1), CYFIP interaction with retrolinkin (TMEM108)^58^, and ABI1 binding to the PSD protein SHANK3^50^ also contribute to the orchestration of WAVE complex activity locally during axonal and dendritic outgrowth or synaptic remodeling (Fig. 2).

## The WAVE complex mediates various cellular events in the brain

### Synapse formation and plasticity

**Fig. 4:**
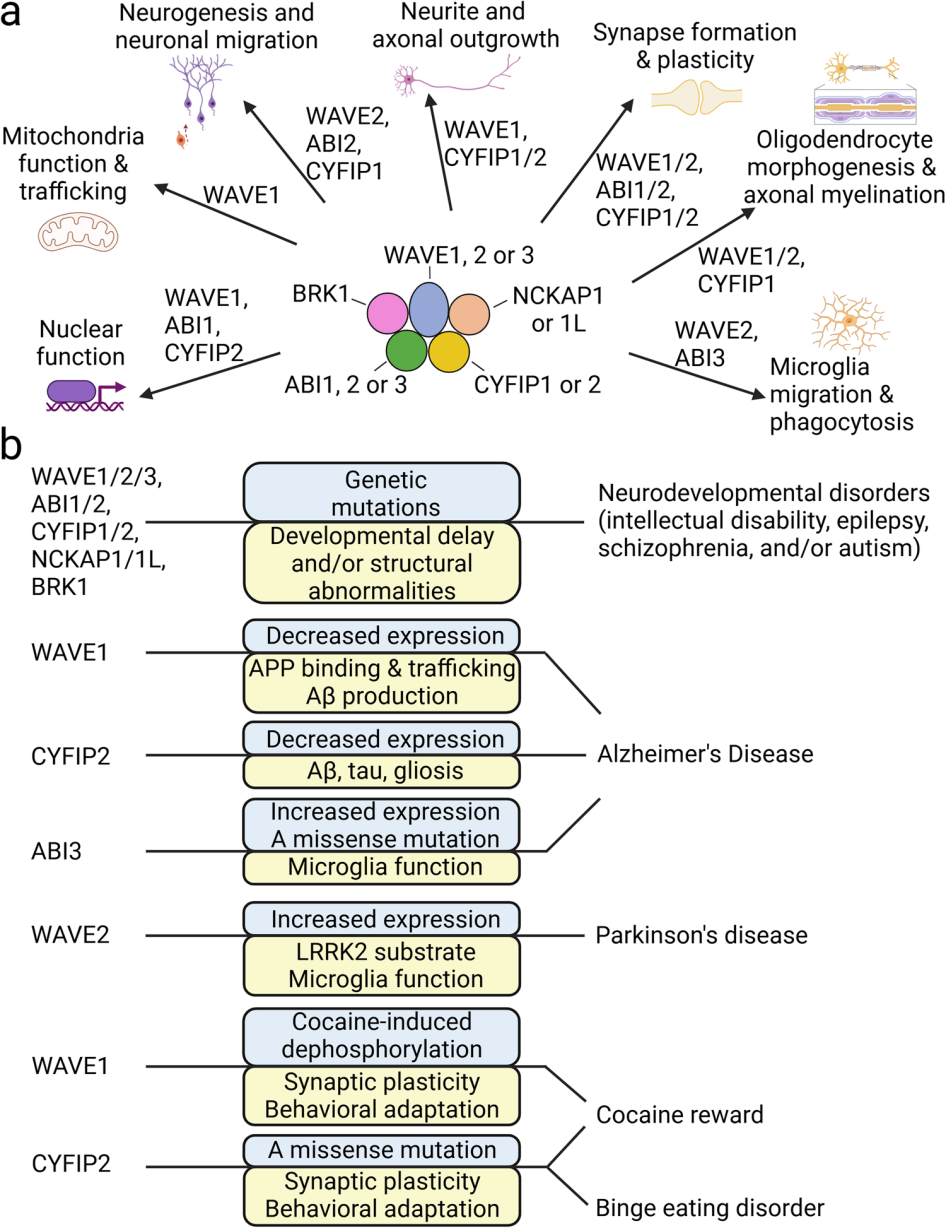
The WAVE complex mediates various cellular events, and alterations in the WAVE complex are associated with brain disorders. **a** Subunit proteins in the WAVE complex are known to be involved in specific cellular events. **b** Alterations (blue box) in specific subunit proteins in the WAVE complex are associated with the pathophysiology (yellow box) of NDDs, AD, PD, cocaine reward and binge eating behavior. This figure was created with BioRender.com.

F-actin is highly enriched in dendritic spines, the postsynaptic parts of excitatory synapses^59^ and presynaptic terminals^60^, and the roles of the WAVE complex in these synaptic compartments have been reviewed in depth^23^ (Fig. 4a). The WAVE1 complex is localized to dendritic spines and plays an important role in the regulation of dendritic spine morphology. WAVE1 deletion decreases the density and alters the morphology of dendritic spines^22,61^. Experimentally decreasing WAVE1 expression causes a decrease in the density of mature dendritic spines with a larger head and shorter neck but increases the density of filopodial dendritic protrusions^22^. WAVE1, a downstream effector of NMDA receptor signaling^44^, is a critical mediator of cognitive function. GluN1 knockdown (GluN1KD) mice have reduced NMDA receptor levels, striatal spine density deficits, and cognitive impairments. Striatal levels of RAC1 pathway components are reduced in GluN1KD mice, with RAC1 and WAVE1 deficits observed at 6 and 12 weeks of age. Concurrently, medium spiny neuron (MSN) spine density deficits are present in mice of these ages. A cross of GluN1KD mice with WAVE1-overexpressing mice rescued striatal WAVE1 protein levels and the MSN spine density, as well as behaviors in the Y-maze and 8-arm radial maze tests^62^. WAVE2 also regulates the dendritic spine density and morphology together with IRSp53^63^ or DYSBINDIN-1^64^, and ABI1 interacts with a proline-rich region of SHANK3 via its SH3 domain and regulates synapse formation^50^. Because genetic variants in DYSBINDIN-1 are associated with schizophrenia (SCZ), the interaction of DYSBINDIN-1 with WAVE2 and ABI1 and their involvement in dendritic spine regulation might be relevant to the pathophysiology of SCZ^50,64^.

CYFIP1 and 2 are enriched in the dendritic spines of excitatory synapses and are involved in the maintenance of dendritic complexity and dendritic spines^65,66^. An increase in the copy number of CYFIP1 is linked to neurodevelopmental alterations. CYFIP1-overexpressing neurons and transgenic mice overexpressing CYFIP1 display immature dendritic spines^67,68^. CYFIP1 and 2 are also expressed in inhibitory synapses^67^. The overexpression of CYFIP1 in cultured hippocampal neurons increases the mEPSC frequency and decreases the mIPSC amplitude. In contrast, excitatory neuron-specific CYFIP1 deletion in mice increases the levels of the neuroligin 3 and GABA_A_ receptor β2/3 subunits and increases inhibitory postsynaptic clustering and mIPSC^67^. The imbalance of excitatory and inhibitory synaptic function likely contributes to the pathophysiology of CYFIP mutations or copy number variations associated with autism and SCZ.

Local mRNA translation in neuronal dendrites is important for synaptic plasticity and brain development. CYFIP1 directly binds to fragile X messenger ribonucleoprotein 1 (FMR1 or FMRP) and mediates the repressive activity of FMRP in dendritic protein translation. CYFIP1/Sra1 directly binds to eIF4E (eukaryotic translation initiation factor 4E) and thereby inhibits translational initiation^69^. Importantly, BDNF or DHPG, a Group I metabotropic glutamate receptor agonist, causes the dissociation of CYFIP1 from eIF4E, suggesting a pivotal role of the FMRP-CYFIP1 complex in neuronal activity-induced local protein synthesis^69^. Thus, CYFIP1 is involved in distinct FMRP-CYFIP1-eIF4E and WAVE complex functions and thereby coordinates local protein translation and actin polymerization in dendritic spines^66,69,70^. BDNF stimulation releases CYFIP1 from eIF4E through RAC1 activation but stabilizes and activates CYFIP1 in the WAVE complex^66^. Thus, the BDNF-RAC1-CYFIP1 pathway can increase both the local protein translation and actin polymerization required for synaptic plasticity^66,69^.

### Neurite and axonal outgrowth

WAVE1 also regulates growth cone dynamics and neurite outgrowth in cultured neurons^61^ (Fig. 4a). WAVE1, together with the RAC GEF Dock3, were found to be downstream effectors of the BDNF-TRKB signaling cascade during axonal outgrowth^71^. Retrolinkin is a regulator of endocytic trafficking of the BDNF-TRKB complex. Retrolinkin interacts with CYFIP2 in the WAVE1 complex and facilitates BDNF-induced TRKB endocytosis and axonal outgrowth^58^. The WAVE1 complex interacts with NYAP (Neuronal tYrosine‐phosphorylated Adaptor for the PI 3‐kinase) family proteins and is involved in PI3K-mediated neurite outgrowth and neuronal morphogenesis^72^. CRMP-2 plays a critical role in axon outgrowth and axon-dendrite specification. CRMP-2 interacts with both the WAVE1 complex and kinesin-1. CRMP-2 and kinesin 1 are required for WAVE1 localization in growth cones, and WAVE1 and CYFIP1 are necessary for CRMP-2-mediated axon outgrowth and axon–dendrite specification^56^. CYFIP1 plays a role in determining the presynaptic terminal size and vesicle release probability during development^73^. CYFIP2 regulates presynaptic functions presumably via the regulation of presynaptic mitochondria number in the adult brain^74^.

### Neurogenesis and neuronal migration

CYFIP1 is required for neural stem cell activity in the subventricular zone and maintenance of the neurogenic niche of the lateral ventricles and thereby cortical neurogenesis in adult mice^75^ (Fig. 4a). CYFIP1 also regulates adult hippocampal neurogenesis via cell-autonomous and non-cell-autonomous mechanisms^76^. Haploinsufficiency of mouse *Cyfip1* results in increased numbers of adult-born hippocampal neurons due to reduced apoptosis without altering proliferation via a failure of microglia to induce apoptosis through the secretion of appropriate factors^76^. In addition, haploinsufficiency of *Cyfip1* causes abnormal migration of adult-born neurons due to altered ARP2/3-mediated actin dynamics^76^. The WAVE2-ABI2 complex is involved in glia-guided radial migration of neurons^77^. The WAVE2-ABI2 complex, which controls growth cone activity, is crucial for completing the transition from a multipolar to a bipolar shape prior to the initiation of glia-guided migration. These growth cone activities are regulated by ABL and CDK5 through the phosphorylation of Tyr150 and Ser137 of WAVE2. The WAVE2-ABI2 complex is therefore important for determining the final location of migrating neurons in the neocortex.

### Oligodendrocyte morphogenesis and axonal myelination

ARP2/3 complex-mediated actin polymerization is important for the initial phase of oligodendrocyte extension and axonal ensheathment, but rapid myelin wrapping is initiated by F-actin disassembly^4,78^. WAVE1 is highly expressed in oligodendrocytes^13^. Compared with WT oligodendrocytes, primary cultured oligodendrocytes isolated from WAVE1 knockout (KO) mice exhibit fewer processes. Hypomyelination in the corpus callosum and a defect in myelin formation in optic nerves in vivo were also observed in WAVE1 KO mice, clearly indicating roles for WAVE1 in oligodendrocyte development and myelination^13^ (Fig. 4a). The *CYFIP1* gene is a candidate risk factor for psychopathology associated with the 15q11.2 BP1-BP2 copy number variant (CNV). Extensive white matter changes, including thinning of the myelin sheath in the corpus callosum of rats or mice with haploinsufficiency of *Cyfip1*, have been observed, suggesting a potential contribution of myelination defects to the increased risk of psychiatric disorders observed in individuals with a 15q11.2 copy number deletion^79,80^.

### Mitochondrial function and trafficking

WAVE1 was identified as a component of a mitochondria-associated complex containing PKA, protein phosphatase-1, the proapoptotic protein BAD and glucokinase in the liver^81^. Immunoelectron microscopy revealed WAVE1 localization on the outer membrane of mitochondria^81^. In addition, WAVE1 was identified in a screen for A kinase-anchoring proteins (AKAPs) and was characterized as a multikinase-scaffolding complex that includes PKA or ABL^82^. In the brain, focal ischemic stroke induces the formation of a protein complex of WAVE1, pancortin-2 and the antiapoptotic protein Bcl-xL. This protein complex is associated with mitochondria and recruits Bax, which is involved in ischemic stroke-induced apoptosis^83^. In primary cultured neurons, WAVE1-mediated actin polymerization is involved in neuronal activity-induced mitochondrial trafficking and fission^45^ (Fig. 4a). Because mitochondrial function is critical for the development of neurons and synapse formation and plasticity, WAVE1-mediated mitochondrial changes may contribute to WAVE1-dependent synaptic changes.

### Nuclear function

Treatment of cells with leptomycin B (an inhibitor of the nuclear exporter CRM-1) induces the nuclear accumulation of ABI1^84^ and CYFIP2^85^. Upon NMDA application, ABI1 translocates from PSDs to nuclei in primary cultured hippocampal neurons and in pyramidal neurons in acute rat brain slices^50^. In the nucleus, ABI1 interacts with the transcription factor complex of Myc/Max proteins and enhances E-box-regulated gene transcription^50^. Xenopus and Drosophila studies also provide insights into the nuclear function of the WAVE complex. Nuclear WAVE1 was identified as a central regulator of the transcriptional reprogramming required for Xenopus oocyte development^86^. WAVE1 is present in the oocyte nucleus. WAVE1 knockdown in embryos results in defective *Hox* gene activation and abnormal development. The role of nuclear WAVE1 in gene activation during development is likely mediated by its WAVE homology domain (WHD), indicating the potential involvement of other subunits of the WAVE complex in nuclear programming^86^. Drosophila WAVE, Scar, and ARP2/3-dependent nuclear actin polymerization and myosin are also critical for heterochromatin repair and stability^87^. Notably, specific nuclear events and gene transcription are regulated by the availability of monomeric actin, since various nuclear proteins, such as chromatin remodeling complexes^88^, histone modifiers^89^ and RNA polymerases^90,91^, require monomeric actin for their activity. In contrast, when double strands of DNA break, nuclear F-actin is required for the non-homologous end joining and homologous recombination repair pathways^92^. Thus, WAVE complex-mediated actin polymerization might control the availability of globular actin (G-actin) or F-actin, which is required for nuclear programming or DNA repair. The roles of WAVE complex-mediated nuclear pathways in brain development, function and disorders remain to be further explored (Fig. 4a).

## The WAVE complex in neurodevelopmental disorders (NDDs)

### *WASF1* (WAVE1 gene) variants

WAVE1 dysfunction is associated with various NDDs, presenting as a broad phenotypic spectrum ranging from mild-to-profound intellectual disability (ID), autism spectrum disorder (ASD), epilepsy, and developmental delay (DD). The loss of WAVE1 results in sensorimotor retardation and diminished learning and memory abilities in mice^10^. Next-generation sequencing has revealed ten distinct sequence variants within *WASF1*. Ito et al. first identified three de novo heterozygous truncating variants clustered around the C-terminal WH2 domain in five unrelated individuals with overlapping neurodevelopmental phenotypes, including severe ID with autistic features and seizures^93^ (Table 2a). Among two nonsense variants and one frameshift variant, the recurrent variant p.Arg506Ter has been identified in three individuals^94–97^. Another nonsense variant, p.Gln520Ter, has also been reported in one individual^98,99^. For the variants truncating the WAVE1 protein, the authors postulate gain-of-function or dominant-negative function due to the presence of a truncated protein in the cells of affected individuals^93^. Notably, the three truncated variants are associated with a similar phenotype as that observed in *Wasf1* KO mice^10^. Owing to the scarcity of reported loss-of-function (LoF) variants, with only 17 cases (allele frequency: ~7E-7) in the Genome Aggregation Database (gnomAD) from healthy subjects^100^, the *WASF1* gene exhibits exceptionally high intolerance to such mutations. In addition, a probability of LoF intolerance (pLI) score of 0.91 for *WASF1*^93^ suggests that LoF is likely the underlying mechanism of the variant. Two additional putative LoF variants have been reported. A heterozygous deletion due to a CNV spanning exons 8–10 of *WASF1* and encompassing the entire GPR6 gene was identified in monozygotic twins with ID, autism, and epilepsy^95^ (Table 2b). An intragenic heterozygous deletion covering exons 4–11 of *WASF1* was found in an individual with a neurodevelopmental psychiatric disorder^101^ (Table 2c).

**Table 2:** Alterations of the WAVE complex in brain disorders.

Gene/Protein	Mutation/Alteration	Organism/Model	Anticipated functional and pathological changes	Brain disorders	Ref.
*WASF1* (NM_003931.2 / NP_003922.1)	**a** Heterozygous truncating or nonsense variants: p.Ile494MetfsTer23 (c.1482delinsGCCAGG), p.Arg506Ter (c.1516C > T), p.Gln520Ter (c.1558C > T)	Human	The authors postulated gain-of-function or dominant-negative function due to the presence of truncated protein. However, LoF is possibly the underlying mechanism.	NDD	^93–99^
	**b** Heterozygous deletion due to a CNV spanning exons 8–10 of *WASF1* and encompassing the entire gene *GPR6*	Human	LoF	ID, ASD	^95^
	**c** Intragenic heterozygous deletion covering exons 4–11	Human	LoF	neurodevelopmental psychiatric disorder	^101^
	**d** Missense variants: p.Trp161Arg (c.481T > A, c.481T > C), p.Leu455Pro (c.1364T > C), p.Leu535Pro (c.1604T > C), p.Trp161Cys (c.483G > T), p.Lys172Glu (c.514A > G)	Human	N.A.	NDD. ID, DD, epilepsy, microcephaly, and ASD	^95,102–104^
	**e** Nonsense variants: p.Lys46Ter (c.136A > T), p.Glu88Ter (c.262G > T), p.Met165AsnfsTer8 (c.493dupA), and p.Ser489Ter (c.1466C > G)	Human	N.A.	NDD	ClinVar database
	**f** Missense variant: p.Ser440Leu (c.1319C > T)	Human	N.A.	N.A.	ClinVar database
*WASF2* (NM_006990.5/ NP_008921.1)	**g** Missense variants: p.Arg38Gln (c.113G > A), c.494T > C (p.Leu165Pro)	Human	N.A.	ASD, NDD	^103–105^
*WASF3* (NM_006646.6/ NP_006637.2)	**h** A nonsense variant: p.Glu88Ter, c.262G > T	Human	N.A.	NDD	^103,104,106^
*CYFIP1*(NM_014608.6 / NP_055423.1)	**i** CNVs at 15q11.2	Human	Microdeletion, LoF	NDDs including ASD, SCZ, ID, and epilepsy	^109^
	**j** rs4778334 and rs1009153	Human	N.A.	SCZ	^110^
	**k** Compound heterozygous variants: p.Ile476Val and p.Pro742Leu	Human	Reduced CYFIP1 stability due to the mutations in protein domains impairing interactions with NCKAP1 and WAVE1 within the WAVE complex.Likely LoF	DD, ID, ASD, and absence of speech	^111^
	Compound heterozygous variants: p.Ile471Val and p.Pro760 Leu	Fruit fly	Reduced axonal projection and volume likely due to deficits in actin polymerization. Likely LoF.	ASD- and SCZ-like behavior including social and motor deficits	
	**l** Missense variant: rs7170637, p.Gly820Ser (c.2458G > A)	Human	N.A.	ASD	^113^
	**m** Missense variants: p.Asp544Asn (c.1630G > A), p.Met956Arg (c.2867T > G), and p.Ala1025Thr (c.3073G > A)	Human	N.A.	ASD	^114^
	**n** Rare heterozygous disruptive nonsense variant in polygenic pattern: p.Arg624Ter (c.1870C > T)	Human	N.A.	SCZ	^115^
	**o** p.Val305del (c.912_914delGGT)	Human	An amino acid in-frame deletion	West syndrome with infantile ID, epilepsy, and ASD	^116^
*CYFIP2* (NM_001037333.2 / NP_001032410.1)	**p** De novo heterozygous missense variants. p.Arg87Cys (c.259C > T), p.Arg87Leu (c.260G > T), and p.Arg87Pro (c.260G > C)	Human	Reduced protein stability or facilitated degradation, leading to LoF	early-onset epileptic encephalopathy, severe psychomotor delay, ID, craniofacial anomalies, hypotonia, and West syndrome	^118–120^
	**q** [p.Glu1174AspfsTer3 (c.3669+1G > T)], [p.Arg87Cys (c.259C > T), p.Tyr108His (c.322T > C), p.Ala455Pro (c.1363G > C), p.Ile664Met (c.1992C>G), p.Glu665Lys (c.1993G>A), p.Asp724His (c.2170G > C), p.Gln725Arg (c.2174A > G).	Human	Predicted to weaken the interaction with WAVE1 or NCKAP1 in WAVE complex; LoF of CYFIP2 for five missense variants (p.Arg87Cys, p.Ile664Met, p.Glu665Lys, p.Asp724His, and p.Gln725Arg) in the interface of CYFIP2-WAVE1; Increased WAVE1 activation and further augmented Arp2/3-mediated actin polymerization representing a gain-of-function of WAVE1	ID, seizures, and muscular hypotonia	^121^
	**r** p.Arg87Ser (c.259C > A), p.Arg87His (c.260G > A), p.Arg87Cys (c.259C > T), p.Ser258Glnfs2 (c.771dup), p.Met311Thr (c.932T > C), p.Met456Val (c.1366A > G), p.Glu468Asp (c.1404G > C), p.Thr490Met (c.1469C > T), p.Lys501Ter (c.1501A > T), Glu686_Ser744delinsAsp (c.2058-1G > C), p.Tyr690Cys (c.2069A > G), p.Asp724Tyr (c.2170G > T), p.Asp724Gly (c2171A>G), p.Asp724His (c.2170G > C), p.Phe888Ser (c.2663T > C), p.His1206Tyr (c.3616C > T),	Human	LoF	DD, ID, ADHD, behavioral problems, seizure, craniofacial anomalies, digital anomalies, epilepsy, hypsarrhythmia, microcephaly, facial dysmorphism, dysphagia, visual problems, and sleep disturbances	^124^
*NCKAP1* (NM_205842.3 / NP_995314.1)	**s** Nonsense variant: p.Glu1100Ter (c.3298G > T)	Human	N.A.	mild ID, ADHD, macrocephaly, and tall stature	^126^
	**t** Frameshift variants: p.Gly175AlafsTer14 (c.523_524insCA), p.Tyr220IlefsTer9 (c.656_657insT), p.Ile765LeufsTer18 (c.2292del), and p.Lys1081IlefsTer15 (c.3240_3241dup) Nonsense variants: p.Lys266Ter (c.796A > T), p.Arg711Ter (c.2131C > T), p.Arg804Ter (c.2410C > T) in two families, p.Glu1088Ter (c.3262G > T), and p.Glu1100Ter (c.3298G > T) Splice variants: s (c.530+3A > G, c.760-3A > C, c.1899+1G > A, c.2522-3C > G, and c.3198+1G > A), Missense variants: [c.5C > G (p.Ser2Trp), c.1537G > A (p.Ala513Thr), and c.3362C > T (p.Ala1121Val)]. Structural variants: a 240 kb de novo microdeletion and one de novo balanced chromosome inversion	Human	N.A.	NDDs with a core feature of autism	^129^
*NCKAP1L* (NM_005337.5/NP_005328.2)	**u** Intronic variant: c.102+6C > T Missense variants: p.Lys954Asn, c.2862G > C p.Leu1008Phe, c.3022C > T Nonsense variants: p.Arg68Ter, c.202C > T p.Arg1082Ter, c.3244C > T	Human	N.A.	ASD	^104,114,133,134^^,188^
*ABI1* (NM_001178116.2/NP_001171587.1)	**v** p.Arg213Ter, c.367C > T p.Thr195Ile, c.584C > T p.Glu413Ala, c.1238A > C	Human	N.A.	Cardiac defect and NDD Myelomeningocele and ASD	^104,114,135–138^
*ABI2* (NM_001282925.3/NP_001269854.1)	**w** De novo heterozygous missense variants p.Arg373Cys, c.1117C > T and p.Glu202Lys, c.604G > A	Human	N.A.	ASD	^127^
	**x** p.Arg132Ter, c.394C > T	Human	homozygous LoF mutation	non-syndromic ID	^140^
*ABI3* (NM_016428.3 / NP_057512.2)	**y** Missense variant: p.Ser209Phe, c.626C > T (rs616338)	Human	impairs overall ABI3 phosphorylation leading to LoF	elevated risk of late-onset AD.	^160^
*BRK1* (NM_018462.5/ NP_060932.2)	**z** Initiation codon variant: p.Met1?, c.2T > A Regulatory sequence variant: c.*135T > C	Human	N.A.	ASD	^114^
*Cyfip2*	Ser968Phe	Mouse-cocaine, amphetamine or food consumption	Destabilization of Cyfip2; Low acute and sensitized response to psychostimulants	Psychostimulant reward Binge eating	^171,173,177^
WAVE1	Dephosphorylation	Mouse-cocaine	Synaptic plasticity and behavioral adaptation	Cocaine reward	^11^
*WASF1*	Low expression	Human AD brain	N.A.	AD	^143,145^
WAVE1	Low expression	AD mice, WAVE1 KO	Reduced Aβ production, improved memory	AD	^143^
CYFIP2	Lower expression	Human AD brain	N.A.	AD	^153,155^
*Cyfip2*	N.A.	KO mice	Aβ accumulation, Tau hyperphosphorylation		
CYFIP1 & CYFIP2	Higher expression	Human	N.A.	FTLD with TDP43	^154^
ABI3	High expression	Human AD brain	N.A.	AD	^162,165^
*Abi3*	N.A.	AD mice, Abi3 KO	Aβ accumulation, reduced LTP, increased inflammation	AD	^164,165^
Scar	Genetic interaction with hLRRK2 (I2020T)	Drosophila	Scar is involved in hLRRK2-induced eye degeneration and dopamine neuronal loss.	N.A.	^168^
WAVE2	Increased WAVE2 expression in LRRK2-G2019S; Decreased WAVE2 expression in *Lrrk2* KO	Human and Mouse models	LRRK2 phosphorylates at T470 of WAVE2 and stabilizes WAVE1 protein.	PD	^169^
	N.A.	Knockdown (mice, drosophila)	Amelioration of LRRK2-G2019S-induced dopamine neuron loss		

*N.A.* not available, *CNV* copy number variant, *ClinVar database* www.ncbi.nlm.nih.gov/clinvar/, *NDD* neurodevelopmental disorders, *LoF* loss-of-function, *ID* intellectual disability, *ASD* autism spectrum disorder, *DD* developmental delay, *SCZ* schizophrenia, *ADHD* attention-deficit/hyperactivity disorder, *AD* Alzheimer’s disease, *FTLD* frontotemporal lobar degeneration, *TDP43* TAR DNA-Binding Protein 43, *PD* Parkinson’s disease.

The remaining five heterozygous missense variants have been reported in patients with NDDs (Table 2d). The phenotypes of two independent patients, each carrying the p.Trp161Arg variant in *WASF1* resulting from distinct genomic alterations—either c.481T > A^95^ or c.481T > C^102^—are similar, displaying ID, DD, epilepsy, and microcephaly. The recurrent variants p.Leu455Pro and p.Leu535Pro were detected in a patient with autism and another patient with DD, respectively^103,104^. Additionally, the variant p.Trp161Cys was reported in a patient with ID, autism, hypotonia, and epilepsy, whereas p.Lys172Glu was found in a patient with DD, hypotonia, and autism^95^. In addition to its marked intolerance toward truncating variants, *WASF1*, with a missense *Z* score of 2.99, is also predicted to be intolerant to missense variants. No discernible phenotypic differences have been observed between LoF variants and missense variants, indicating that the mutated residues are located within functionally crucial domains. Trp161 and Lys172 reside within the WHD (also called WH1, WASP homology 1, domain) and basic region, respectively^95^, whereas Leu535 is located within the WH2 domain. In the ClinVar database, four more nonsense variants associated with NDD were listed: p.Lys46Ter, p.Glu88Ter, p.Met165AsnfsTer8, and p.Ser489Ter (Table 2e). Additionally, one missense variant, p.Ser440Leu, is included, for which no phenotypic information is available (Table 2f). Interestingly, microcephaly, which is not observed in KO mice, manifested in two monozygotic twins with a deletion spanning the entire *GPR6* gene and exons 8–10 of *WASF1*^95^, as well as in two independent patients harboring the identical missense variant p.Trp161Arg^95,102^. However, this phenotype was not observed in any other patients with *WASF1* mutation.

### *WASF2* variants

WAVE2 is expressed at relatively high levels in microglia, perivascular macrophages and vascular endothelial cells in the brain (Fig. 1b and Table 1). Two missense *WASF2* variants, c.113G > A (p.Arg38Gln) and c.494T > C (p.Leu165Pro), were identified in patients with autism and NDDs^103–105^, but the functional alterations associated with brain disorders remain to be studied (Table 2g).

### *WASF3* variants

Although WAVE3 is expressed in neuronal cells, particularly GABAergic interneurons (Fig. 1b and Table 1), its function is less well characterized than that of WAVE1. A nonsense *WASF3* variant, p.Glu88Ter, was identified in a patient with NDDs^103,104,106^ (Table 2h).

### *CYFIP1* variants

CYFIP1 has been implicated in autism^107,108^. CNVs in the 15q11.2 region are found in patients with NDDs, including ASD, SCZ, ID, and epilepsy (Table 2i). The smallest region linked to this condition spans between breakpoints BP1 and BP2 on chromosome 15q11.2. Among the four genes (*TUBGCP5, CYFIP1, NIPA2*, and *NIPA1*) encompassed within this narrow region, increasing evidence highlights the relevance of *CYFIP1*^109^.

Following the association of two common SNPs in *CYFIP1* in SCZ patients^110^ (Table 2j), the compound heterozygous variants p.Ile476Val and p.Pro742Leu were identified via exome sequencing in two affected males from a family, who exhibited DD, ID, ASD, epilepsy, and an absence of speech (Table 2k). These mutations occur in protein domains responsible for maintaining interactions within the WAVE complex. Transgenic fly models with these mutations presented an abnormal neuronal morphology and F-actin loss, mirroring core behavioral symptoms such as social interaction and motor coordination deficits. Molecular and cellular analyses of skin fibroblasts from one proband revealed actin polymerization deficits, potentially due to impaired WAVE complex functionality and reduced CYFIP1 stability within the complex^111^.

Dysregulation of protein synthesis in neurons has been implicated in the pathogenesis of various NDDs^112^. In a subgroup of fragile X syndrome (FXS) patients exhibiting severe ASD and obsessive‒compulsive behavior, the *CYFIP1* mRNA is downregulated, particularly in patients with the Prader‒Willi phenotype^108^. *CYFIP1* downregulation or genetic alterations in *CYFIP1* might alter the FMRP-CYFIP1-eIF4E-mediated repression of local protein synthesis in neurons^69^. Four heterozygous missense *CYFIP1* variants were identified in patients with ASD, including a common variant, p.Gly820Ser^113^ (Table 2l), and three rare variants, p.Asp544Asn, p.Met956Arg and p.Ala1025Thr^114^ (Table 2m). Additionally, a polygenic pattern including a rare heterozygous disruptive nonsense variant, p.Arg624Ter, in CYFIP1 was detected in a patient diagnosed with SCZ^115^ (Table 2n). Furthermore, an amino acid in-frame deletion, p.Val305del, was identified in a patient with West syndrome, who was characterized by infantile ID, epilepsy, and ASD^116^ (Table 2o).

### *CYFIP2* (*PIR121*) variants

Like CYFIP1, CYFIP2 is present at both excitatory and inhibitory synapses, where it interacts with both the WAVE complex and FMRP^117^. Three de novo heterozygous missense variants in *CYFIP2* were identified in four unrelated individuals diagnosed with early-onset epileptic encephalopathy through trio whole-exome sequencing (WES), suggesting the association of this gene with NDDs. All three variants affect Arg87—p.Arg87Cys, p.Arg87Leu, and p.Arg87Pro—suggesting that it represents a hotspot for these mutations (Table 2p). The accompanying phenotypes include severe psychomotor delay, ID, craniofacial anomalies, and hypotonia^118^. Interestingly, West syndrome was diagnosed in two individuals^118^, similar to the reported patient with a *CYFIP1* variant^116^. Based on the LoF intolerance score (pLI = 1) of *CYFIP2*, the authors initially assumed that variants in Arg87 might lead to impaired WAVE-mediated actin dynamics through an LoF mechanism, but subsequent protein modeling and functional analyses suggest a model of sustained activation of the WAVE complex by Arg87 variants^118^. However, the LoF mechanism of CYFIP2 was further supported by the finding of protein instability of CYFIP2 variants in cells^119^ and in the brain^120^. A knock-in mouse model of the p.Arg87Cys variant recapitulated many neurological and neurobehavioral phenotypes found in patients with this variant^120^. In addition, disorganization of the neuronal layers and gliosis in the brain were observed in the knock-in mice. The Arg87Cys variant enhances the ubiquitination and proteasomal degradation of CYFIP2 in cultured cells and mouse brains, establishing an LoF mechanism involving Arg87 variants^120^. Using trio next-generation sequencing, a total of eight distinct de novo variants in *CYFIP2* were identified among 12 independent patients who shared a phenotype characterized by ID, seizures, and muscular hypotonia. One is a splice variant, while the remaining seven are missense variants (Table 2q). These variants spatially cluster in the tertiary structure and are all predicted to weaken the interaction with WAVE1 or NCKAP1 in the WAVE complex^121^.

*Cyfip2* homozygous deletion is lethal in mice, indicating the vital role of CYFIP2 in early development, whereas heterozygous KO mice exhibit abnormal behaviors and cortical dendritic spines, similar to those observed in *Fmr1*-null mice^122^. Therefore, along with the LoF intolerance score of *CYFIP2* (pLI = 1), the authors initially assumed that haploinsufficiency of *CYFIP2* causes the neurodevelopmental phenotype in humans. However, the phenotype in the heterozygous *Cyfip2* KO mouse model was milder than the severe phenotype observed in humans heterozygous for variants^121^. In genes linked to autosomal dominant diseases, heterozygous mutations are sufficient to manifest the phenotype in humans. In comparison, heterozygous KO mice present no obvious phenotype, whereas homozygous KO mice present a phenotype similar to that of humans^123^. Zweier et al. further hypothesized that the detected variants promote a partial LoF of *CYFIP2*, particularly the five missense variants (p.Arg87Cys, p.Ile664Met, p.Glu665Lys, p.Asp724His, and p.Gln725Arg), which are located close to the CYFIP2‒WAVE1 interface and are predicted to impair the binding of the WAVE WCA region and the release of the WCA for ARP2/3-mediated actin polymerization, suggesting a gain-of-function scenario^121^.

Begemann et al. reported 19 additional individuals harboring *CYFIP2* variants through exome sequencing, including 14 with novel variants (Table 2r). Five individuals had two reported variants, p.Arg87Cys and p.Asp724His, with p.Asp724 being the second mutational hotspot. Among them, three individuals harboring LoF variants—p.Lys501Ter, p.Ser258Glnfs2, and c.2058-1G > C—exhibited clinical features such as DD, ID, attention-deficit/hyperactivity disorder (ADHD), behavioral problems, seizures, craniofacial anomalies, and digital anomalies. The phenotypes of individuals with missense variants included DD, ID, epilepsy, hypsarrhythmia, microcephaly, facial dysmorphism, dysphagia, visual problems, and sleep disturbances. The authors argued that patients with LoF variants did not present phenotypes as severe as those observed in individuals with missense variants^124^. However, *CYFIP2* is intolerant to both missense (z = 6.01) and LoF variants (pLI = 1). Additionally, pathogenic variants within the same neurodevelopmental gene may result in diverse clinical presentations, including differences in severity^125^. Consequently, these mutations likely lead to the LoF of *CYFIP2*.

### *NCKAP1* (*HEM2*) variants

A nonsense variant (p.Glu1100Ter) of *NCKAP1* was identified as segregating within a multi-generational family comprising 13 affected members exhibiting mild ID, ADHD, macrocephaly, and tall stature (Table 2s). This variant is expressed in cells in diverse brain regions, including Purkinje cells, the dentate nucleus of the cerebellum, the CA4 region, and the dentate gyrus of the hippocampus, and frontal gray and white matter regions^126^.

In the Simons Simplex Collection (SSC), two de novo truncating mutations in *NCKAP1*-p.Gly175TrpfsTer14 and p.Glu1088Ter- were identified in two ASD probands from two unrelated simplex quad families^127^. An analysis of genome sequencing data from 2,308 individuals in 493 multiplex ASD families from the Autism Genetic Resource Exchange (AGRE) conducted through TADA (Transmitted And De novo Association) mega-analysis identified *NCKAP1* among 69 genes associated with an ASD risk^128^.

A total of 19 predicted deleterious variants in *NCKAP1* were reported across 20 unrelated families. These variants include four frameshift variants, five nonsense variants, five splice variants, and three missense variants (Table 2t). Additionally, a 240 kb de novo microdeletion encompassing three genes, including *NCKAP1* at 2q32.1, and one de novo balanced chromosome inversion [46,XX,inv(2)(p23.1q32.1)dn] truncating *NCKAP1* at the 2q32.1 genomic inversion breakpoint were identified. Among them, 13 were found to be de novo, four were transmitted, and three had an unknown inheritance pattern^129^.

### *NCKAP1L* (*HEM1*) variants

NCKAP1L is hematopoietic cell-specific and is expressed selectively in microglia in the brain (Fig. 1e and Table 1). NCKAP1L mutations in humans and HEM1 deletion in mice cause broad immune dysregulation^130–132^. Next-generation sequencing identified one intronic^114^, two missense^133,134^, and two nonsense variants in autism patients^104,114^, and the functional consequences of these variants remain to be established (Table 2u).

### *ABI1* variants

A nonsense variant, c.367C > T, p.Arg213Ter, in *ABI1* was identified in a patient with cardiovascular dysfunction and NDD^135–137^ (Table 2v). Additionally, two missense variants, c.584C > T, p.Thr195Ile, and c.1238A > C, p.Glu413Ala, in *ABI1* were found in patients with myelomeningocele (spina bifida)^138^ and ASD^104,114^, respectively. *Abi1*-null mouse embryos are smaller, with some unturned embryos and undulation of the neural tube as early as E8.5^139^. In some embryos, the neural tube was open. *Abi1*-null mouse embryos exhibited lethality between E10.5 and E11.5^139^. *Abi1*-KO cells displayed defective regulation of the actin cytoskeleton, which was attributed to altered activity of the WAVE1 and 2 complexes. Compared with those in control cells, the levels of the WAVE complex components WAVE1/2, CYFIP1/2, and NCKAP1 were significantly lower, but the ABI2 protein levels in *Abi1*-KO cells were twofold higher^139^.

### *ABI2* variants

Two de novo heterozygous missense mutations, ABI2-p.Arg373Cys and p.Glu202Lys, were identified in two ASD probands from two independent simplex quad families through exome sequencing data retrieved from the Simons Simplex Collection (SSC)^127^ (Table 2w). Furthermore, a homozygous LoF mutation, p.Arg132Ter, in *ABI2* was identified through a combination of microarray genotyping, homozygosity-by-descent (HBD) mapping, CNV analysis, and exome sequencing. This discovery was made within a cohort of 192 multiplex Pakistani and Iranian consanguineous families. *ABI2* is one of the 26 newly identified candidate genes associated with autosomal recessive non-syndromic ID^140^ (Table 2x).

The homozygous deletion of murine *Abi2* led to abnormal phenotypes in the eye and brain, which are the tissues with the highest ABI2 expression. Additionally, the loss of ABI2 resulted in defects in cell migration within the neocortex and hippocampus, as well as abnormalities in dendritic spine morphology and density. These disruptions are accompanied by severe deficits in both short- and long-term memory^141^.

### *ABI3* variants

NESH (**NE**w molecule containing **SH**3) was initially isolated from a human placental cDNA library as a novel gene featuring an SH3 domain, along with proline-rich and serine-rich regions. Due to its sequence similarity to ABI1 and ABI2, it was subsequently incorporated into the ABI family and designated ABI3^142^. An ABI3 mutation associated with AD (Table 2y) is discussed below.

### *BRK1* (*HSPC300*) variants

An initiation codon variant c.2T > A, p.Met1? and a regulatory sequence variant, c. *135T > C in BRK1 was identified in two patients with autism spectrum disorder^114^, but the functional consequences of the variants remain to be studied (Table 2z).

## The role of the WAVE complex in neurodegenerative diseases

### AD

Alterations of the WAVE complex have been implicated in the pathophysiology of AD. A decrease in *WASF1* (human WAVE1 gene) expression was observed in human AD brains compared with healthy control brains and in mouse models of AD compared with controls^143^. Downregulation of *WASF1* expression in the AD brain has also been observed in publicly available datasets^144,145^. A likely mechanism supported by the experimental data is that the AICD (APP intracellular domain) generated from the amyloidogenic pathway binds to the WAVE1 promoter and negatively regulates its transcription and protein expression^143^. On the other hand, WAVE1 interacts with and colocalizes with APP in the Golgi apparatus. Experimentally reducing WAVE1 expression decreases the budding of APP-containing vesicles from the Golgi and reduces the trafficking of APP to the cell surface, thereby inhibiting Aβ production^143^. WAVE1 KO dramatically reduces Aβ levels and restores memory deficits in APP/PS1 transgenic mice expressing familial mutations in *APP* (APPswe) and presenilin 1 (*PSEN1* ΔE9)^146^, suggesting a protective role for reduced WAVE1 expression in AD^143^. The colocalization of the WAVE1 protein or phosphorylated WAVE1 with aggregated hyperphosphorylated tau was observed in the brains of triple transgenic mice (APPswe/PS1M146V/tauP301L)^47^ and human AD patients^147^. The WAVE1 gene has also been suggested as an AD-associated hub gene from studies of AD mouse models^148^ or human AD brains^149^.

Compared with that in non-demented controls, the level of RAC1 in the frontal cortex of AD patients is decreased, but it is transiently decreased in the brains of an animal model of AD (3xTg, 7 months)^150^. The activation of RAC1 in a cell line or primary cultured cortical neurons increased Aβ levels and tau hyperphosphorylation. Inhibition of RAC1 attenuated γ-secretase activity for APP^151^. *NCKAP1* expression was also discovered to be markedly reduced in the brains of AD patients compared with those of normal subjects^152^, but the functional roles of NCKAP1 downregulation in AD remain to be established.

Reduced expression of CYFIP2 was observed in postmortem AD brains and in a mouse model of AD^153^. CYFIP1 is also decreased in individuals with late-stage AD, but the protein levels of both CYFIP1 and CYFIP2 are increased in the postmortem hippocampal tissues of patients with frontotemporal lobar degeneration (FTLD) with TDP43 pathology^154^. Several AD-like pathologies, including Aβ accumulation, tau hyperphosphorylation, gliosis and dendritic spine loss in the hippocampus, have been observed in heterozygous *Cyfip2* KO mice^153,155^ but not in mice with a neuron-specific conditional KO of *Cyfip2*^156^. Researchers have not clearly determined why the effect of CYFIP2 KO on AD-like pathologies is detrimental, although the effect of WAVE1 KO on amyloid pathology is protective^143^. These opposing results might be due to the inhibitory action of CYFIP2 on WAVE1-mediated actin polymerization^118,157^ or the involvement of CYFIP2 in mRNA translation^155^.

Human genetics studies of AD identified a coding variant of *ABI3*, p.Ser209Phe (rs616338), associated with late-onset AD^158–161^ (Table 2y). The presence of Phe209 impairs overall ABI3 phosphorylation, rendering the ABI3 protein functionally inactive. This alteration could be interpreted as causing hypomorphic function or partial LoF^53^. ABI3 is highly expressed in microglia^162,163^ (Fig. 1c and Table 1), and ABI3 expression is increased in postmortem AD brains^162,164^. The deletion of *Abi3* in mice accelerates Aβ accumulation, decreases microglial migration and clustering around plaques, and reduces LTP due to Aβ accumulation in a mouse model of AD (5XFAD)^164,165^. In addition, *Abi3* KO also increases body and fat mass and reduces energy expenditure^166^. Due to the genetic overlap between *Abi3* and *Gngt2* on chromosome 11q in mice (not present in humans at 17q21.32 with a reverse transcription direction), *Abi3* KO also targets *Gngt2*^53^. *Abi3*-*Gngt2* dual KO predisposes mice to an immune phenotype characterized by reactive gliosis and gene expression patterns resembling those associated with AD. Notably, the AD-associated genes *Trem2*, *Plcg2*, *Tyrobp*, and *Csf1r* are upregulated in a gene dose-dependent manner in these mice, even in the absence of Aβ accumulation. In APP transgenic TgCRND8 mice, loss of *Abi3*-*Gngt2* results in a gene dose- and age-dependent reduction in Aβ deposition, but in *Abi3*-*Gngt2*^−/−^ mice, AAV-mediated expression of a pro-aggregating form of human tau exacerbates tauopathy and astrocytosis^53^.

### Parkinson’s disease (PD)

Leucine-rich repeat kinase 2 (LRRK2) is the most common gene linked to PD. *LRRK2* mutations are associated with familial and sporadic PD, and LRRK2 is a promising therapeutic target for PD^167^. Using an hLRRK2-induced eye degeneration model in Drosophila, genetic modifiers of LRRK2 were screened, and Scar/WAVE was found to be a genetic interactor of LRRK2^168^. In BV2 microglia, LRRK2 knockdown reduces the level of WAVE2 but not the levels of WAVE1 and WAVE3. WAVE2 levels are also reduced in primary microglia and bone marrow-derived macrophages from LRRK2 KO mice. However, an increase in WAVE2 expression was observed in microglia and macrophages from LRRK2-G2019S knock-in mice^169^. LRRK2 binds and phosphorylates mouse WAVE2 at Thr470 (Thr471 in human WAVE2, Fig. 3b), stabilizing and preventing the proteasomal degradation of WAVE2, whereas LRRK2 does not interact with WAVE1^169^. Macrophages or microglia isolated from PD patients or LRRK2-G2019S mice display increased phosphorylation at Thr470 on WAVE2 and enhanced phagocytic responses, but *LRRK2* deletion causes the opposite effects. In addition, WAVE2 and its phosphorylation are involved in LRRK2-G2019S-induced dopaminergic neuronal death in macrophage and midbrain cocultures. Furthermore, WAVE2 knockdown in mice and Scar knockdown in Drosophila ameliorate LRRK2-G2019S-induced dopaminergic neuron loss^169^. An LRRK2 kinase inhibitor or an ARP2/3 inhibitor prevents microglial activation and neurotoxicity in vitro^169,170^, suggesting the involvement of the LRRK2-WAVE2 pathway in microglia in PD pathogenesis.

## The role of the WAVE complex in other brain disorders

Psychostimulant drug-induced behavioral responses require WAVE1 complex-mediated synaptic modulation. C57BL/6 mouse substrains have different locomotor activities and sensitized responses to cocaine and methamphetamine. Genetic screening of C57BL/6N mice, which have a low acute and sensitized response to psychostimulants compared with C57BL/6J mice, identified a Ser968Phe mutation in *Cyfip2* as a causative variant^171^. CYFIP2 is destabilized by the Ser968Phe mutation, and deletion of the allele in C57BL/6N mice results in acute and sensitized cocaine response phenotypes, suggesting that CYFIP2 is a critical regulator of the cocaine response^171^. Alternatively, cryo-electron microscopy of the WAVE complex with RAC1 indicated that the Ser968Phe mutation in CYFIP1 (corresponding to Ser968Phe in CYFIP2) is located just beneath the RAC1 binding site. Further characterization revealed that Ser968Phe in CYFIP1 facilitates RAC1 binding and WAVE complex activation^172^. CYFIP2 Ser968Phe knock-in mice display attenuated cocaine-induced behavioral sensitization and conditioned place preference^173^. Cocaine-induced increases in c-Fos and GluA1 expression and an increase in dendritic spine density in the nucleus accumbens, a key brain region for cocaine reward, were observed in wild-type mice but not in CYFIP2 Ser968Phe knock-in mice, establishing the significance of this variant in cocaine responses^173^. Another study of BXD mouse strains suggested that *Cyfip2* is a candidate gene associated with different intake responses in a cocaine self-administration paradigm^174^.

WAVE1 is also required for cocaine-induced behavioral and synaptic plasticity^11^. Cocaine blocks presynaptic dopamine reuptake^175^ and induces dopamine release^176^, thereby increasing synaptic dopamine levels. WAVE1 deletion in neurons expressing the dopamine D1 receptor but not the dopamine D2 receptor significantly decreases cocaine reward, as measured by cocaine-associated place preference and cocaine-induced sensitized locomotor behavior^11^. Cocaine administration also reduces inhibitory phosphorylation at Ser310, 397 and 441 on WAVE1 in an NMDA receptor- or dopamine D1 receptor-dependent manner. WAVE1 is required in neurons expressing the dopamine D1 receptor for cocaine-evoked glutamatergic synaptic plasticity^11^. Together, these results suggest critical roles for WAVE1 and CYFIP2 in synaptic plasticity in the reward regions of the brain, mediating short-term and long-term behavioral responses to psychostimulant drugs.

CYFIP2 was also identified as a significant genetic factor underlying binge eating by analyzing closely related C57BL/6 mouse substrains^177^. C57BL/6N mice, but not C57BL/6J mice, showed rapid and robust escalation in palatable food consumption. *Cyfip2* heterozygous KO mice on a C57BL/6N background presented reduced binge eating comparable to that of wild-type C57BL/6J-like mice. Because CYFIP1 is deleted or imprinted in patients with type I Prader–Willi syndrome, an NDD defined by extreme hyperphagia and a hyperresponsive reward system to food-associated cues, the shared biological functions of CYFIP1 and 2 might be associated with maladaptive feeding in humans^177,178^.

## Current limitations and remaining questions

Despite a variety of compelling results, our understanding of the regulatory mechanisms of the WAVE complex in specific cell types in the brain is incomplete. Previous studies established the interactions between acidic phospholipids, protein activators, membrane-associated or cytoplasmic binding partners and protein kinase- or phosphatase-dependent mechanisms for full WAVE complex activation using in vitro reconstitution assays and structural biology approaches (Fig. 2). Structural studies of the WAVE complex in vitro have used a recombinant WAVE complex containing mini-WAVE^21,28,30,172^, which lacks the proline-rich region due to the disordered nature of this region and difficulty in crystallization^21^. Although WAVE1 exhibits high levels of basal phosphorylation in neurons or the brain^22,49^, this baseline phosphorylation is not present in the purified recombinant WAVE complex. In fact, several studies have used intact WAVE complexes isolated from the brain or cells^19,20,22,179^. The native full-length WAVE2 complex was successfully activated by the cooperative actions of prenylated RAC-GTP, acidic phospholipids and phosphorylation^20^. However, the interpretation of in vitro actin polymerization, especially using the native full-length WAVE1 complex, is sometimes limited, and its regulatory mechanism is still incompletely understood because of the potential artifacts observed under in vitro conditions or the failure to reproduce the activation of the native WAVE1 complex by RAC1 and PIP_3_^19,20^. Phosphorylation site-specific antibodies or measurements of the expression levels of subunits of the WAVE complex have been used to assess alterations in the WAVE complex in the brain or neurons. However, it is not technically feasible to assess WAVE complex activity in vivo, which is affected by multiple regulatory inputs, including protein activators, phospholipids, phosphorylation or dephosphorylation, and membrane-associated interactors (Fig. 2).

Alterations in the regulatory subunits in the WAVE complex likely affect WAVE-mediated actin polymerization. However, because ABI can regulate N-WASP^180^, CYFIP can bind to FMRP and eIF4E^69^ as well as multiple RNA-binding proteins^181^, and NCKAP1L can also interact with mTOR complex 2 via rapamycin-insensitive companion of mTOR (RICTOR)^130^, researchers have not conclusively determined whether the effects of disease-associated variants of CYFIP, NCKAP or ABI are dependent on or independent of WAVE-mediated actin polymerization. Thus, fully understanding whether increased or decreased WAVE complex-mediated actin polymerization contributes to disease progression is important.

The evidence for pivotal contributions of alterations in the WAVE complex to the development of NDDs and neurodegenerative diseases is growing (Fig. 4b). Alterations in WAVE1, ABI1/2 and CYFIP1/2 in neurons or ABI3 and WAVE2 in microglia have been implicated in the pathophysiology of NDDs, AD or PD. The potential contributions of dysfunction of the WAVE complex in other cell types, such as oligodendrocytes and astrocytes, to disease development remain to be investigated. Recent studies have indicated that leukocyte infiltration and aberrant inflammation exert protective or detrimental effects on the development of neurodegenerative diseases^182,183^. Whether WAVE2, NCKAP1L and CYFIP2 function in T cells^130,184,185^; WAVE1 and WAVE2 function in macrophages^186,187^; or WAVE complex-mediated actin polymerization in peripheral and central immune cells is involved in pathogenic or protective mechanisms for brain disorders remains to be explored. An intriguing question is how gain-of-function or loss-of-function of the WAVE complex segregates as a risk factor for apparently distinct developmental versus adulthood or neurodegenerative diseases (Table 2 and Fig. 4b). The generation of in vivo models of WAVE complex variants^120,173^ to characterize the role of functional alterations in the WAVE complex in disease progression will be a critical task in the coming years.
